# Maternal vaccination with live-attenuated Rift Valley fever virus protects offspring via immune transfer

**DOI:** 10.1038/s41541-025-01230-w

**Published:** 2025-07-25

**Authors:** Austin T. Hertel, Cynthia M. McMillen, Ryan M. Hoehl, Dominique J. Barbeau, Anita K. McElroy, Amy L. Hartman

**Affiliations:** 1 Center for Vaccine Research, University of Pittsburgh, Pittsburgh, PA USA; 2 Department of Infectious Disease and Microbiology, School of Public Health, University of Pittsburgh, Pittsburgh, PA USA; 3 Division of Pediatric Infectious Disease, Department of Pediatrics, School of Medicine, University of Pittsburgh, Pittsburgh, PA USA

**Keywords:** Live attenuated vaccines, Viral infection

## Abstract

Rift Valley fever virus (RVFV) causes high rates of spontaneous abortions and neonatal mortality in ruminants resulting in severe socioeconomic and public health consequences. Maternal vaccination may protect pregnant animals, fetuses, and neonates via transfer of maternal antibodies; however, currently available live-attenuated RVFV vaccines are generally unsafe for use during pregnancy. RVFV-delNSs/NSm is a live attenuated strain that has demonstrated favorable safety and efficacy in pregnant livestock, yet studies investigating maternal vaccination as a strategy to protect neonates from RVF are limited. Using pregnant Sprague-Dawley rats, we show that maternal vaccination with RVFV-delNSs/NSm leads to efficient transfer of anti-RVFV antibodies to offspring. These offspring were completely protected from lethal RVFV challenge. Although further investigation is required in susceptible ruminant species, our findings indicate that maternal anti-RVFV immunity is sufficient to protect offspring, highlighting maternal vaccination as a potential strategy to reduce RVF disease burden in endemic regions.

## Introduction

Rift Valley Fever virus (RVFV) is a re-emerging arbovirus endemic to Africa that poses a formidable threat to animal and human health. RVFV has a complex transmission cycle where infected mosquitoes can transmit the virus to both humans and livestock (sheep, goats, cattle, and camels). Livestock serve as amplifying hosts^1,2^, driving spillover into humans in close contact with infected animals^3^. There is also evidence that people living in urban settings with limited livestock exposure are also at risk of infection via consumption of raw milk or other animal products^4–6^. Rift Valley fever (RVF), the disease caused by RVFV, generally presents as a mild febrile illness in humans; however, some individuals will experience severe and even lethal outcomes such as hemorrhagic fever and meningoencephalitis^7^. RVF in animals is distinguished by high rates of spontaneous abortions in pregnant animals and near 100% neonatal mortality^8–10^. This can result in the loss of whole generations of animals which can have severe socioeconomic consequences for the affected regions^11^. Furthermore, livestock trade from endemic countries poses a risk of introducing RVFV to previously unaffected regions^12^. Livestock trade was likely responsible for the emergence of RVFV in the Arabian Peninsula that led to trade bans and millions of dollars in economic losses^13,14^. Overall, uncontrolled RVFV circulation in vulnerable livestock populations, such as pregnant and neonatal animals, increases human disease burden, causes socioeconomic damage, and increases the risk of viral emergence into new geographic regions.

Despite decades of RVFV vaccine research, there are currently no licensed vaccines available for use in humans. Several live-attenuated vaccines (LAVs) such as Smithburn and Clone-13 are approved for veterinary use in endemic countries to control the spread of RVFV^15^. Epidemiological modeling estimates that reactive livestock vaccination campaigns can reduce human RVF cases by as much as 30%^16^. Therefore, livestock vaccination is a cost-effective and efficient strategy to reduce human disease burden in resource-limited regions. A major limitation of existing RVFV LAVs is their partially retained virulence in pregnant animals^17–19^ and lack of serological markers for differentiating infected from vaccinated animals (DIVA). To overcome these challenges, a next-generation RVFV vaccine candidate, RVFV-delNSs/NSm was developed. The vaccine is based on the parental ZH501 strain and includes complete deletions of the two known RVFV virulence genes, the non-structural proteins, NSs and NSm^20^. RVFV-delNSs/NSm vaccination induces robust immunity in a variety of animal species (rats, sheep, and non-human primates) and confers protection against wild-type (WT) virus challenge^20–22^. Additionally, RVFV-delNSs/NSm has demonstrated a high degree of safety in pregnant ewes^21^. Furthermore, DDVax, which is a patented vaccine based upon the genetic deletion of NSs and NSm, is being advanced toward human clinical trials by the Coalition for Epidemic Preparedness Innovations (CEPI)^23^.

Maternal vaccination can protect pregnant animals and their offspring through passive transfer of humoral immunity *in utero* and postnatally via milk^24^. However, studies investigating maternal vaccination as a strategy to protect neonates from RVF during early life are lacking. To address this, we used a Sprague-Dawley rat model to study the transfer of anti-RVFV immunity following maternal vaccination with RVFV-delNSs/NSm during pregnancy. The goal of this study was to assess the kinetics of anti-RVFV immune transfer to offspring following maternal vaccination during pregnancy and to determine if anti-RVFV immunity acquired through milk would protect neonates from WT RVFV challenge. Maternal livestock vaccination could serve as an important strategy to reduce animal infections, human spillover, and subsequent socioeconomic damage exemplifying the One Health concept. While substantial physiologic differences in pregnancy and transfer of maternal immunity between species exist, this rodent model provides a blueprint for evaluating future vaccine approaches in susceptible livestock species.

## Results

### Maternal anti-RVFV immunity is efficiently transferred to offspring via milk

Maternal vaccination during pregnancy can be leveraged to confer protection to neonates via passive antibody transfer in milk. For RVFV, this can be particularly important given the high susceptibility of pregnant animals and newborns to lethal disease. In rodents, most maternal circulating IgG is acquired postnatally via colostrum and milk, with little transferred trans-placentally *in utero*^25^. In livestock such as sheep and cattle, maternal antibodies are obtained exclusively in the post-natal period via colostrum and milk^26^.

**Fig. 1:**
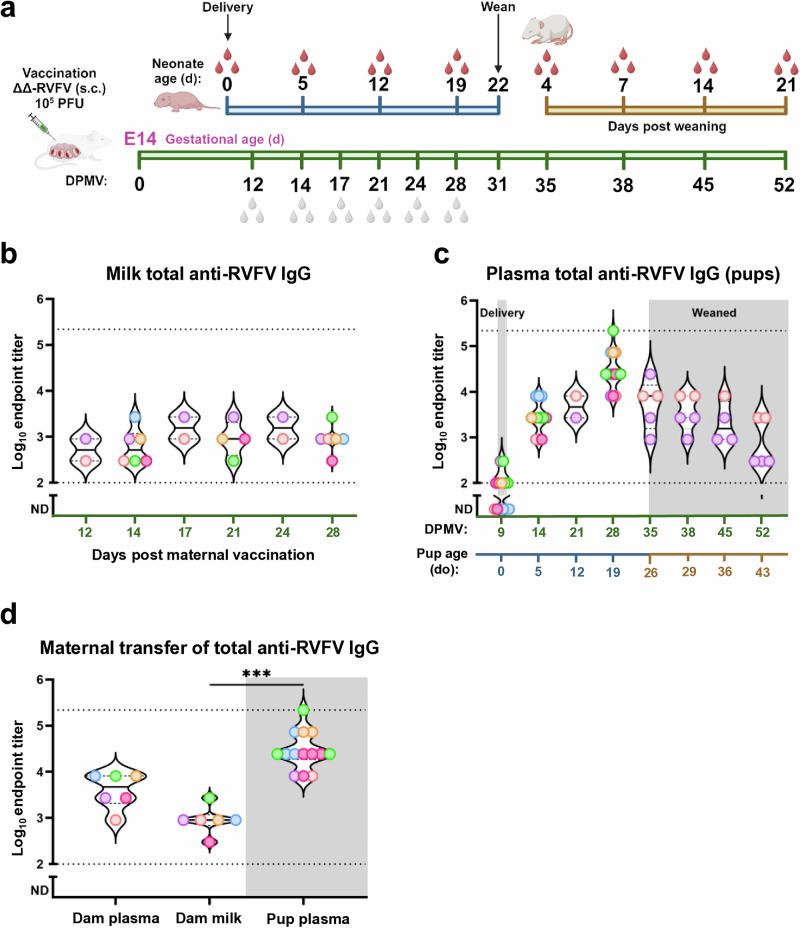
Transfer of anti-RVFV humoral immunity from pregnant dams to neonatal rat pups. **a** Pregnant (E14, *n* = 6) dams were vaccinated s.c. with 1 × 10^5^ PFU RVFV-delNSs/NSm, completed pregnancy, delivered and nursed their pups until the pups reached 22 d.o. (31 d.p.m.v.). For milk, serial samples were taken from each dam (green timeline). For suckling pups (≤22 d.o.), a subset of each litter was euthanized at each timepoint for blood collection (blue timeline). For weaned pups (≥22 d.o.), blood was serially collected from each pup (gold timeline) until the study endpoint (52 d.p.m.v., 43 d.o.). White and red droplets indicate collection timepoints for milk and pup blood, respectively. **b,** **c** Violin plots displaying total anti-RVFV IgG titers in milk (**b**) and pup plasma (**c**) over time measured by ELISA. **d** Violin plots showing total anti-RVFV IgG titers in dam plasma and milk (*n* = 6, 28 d.p.m.v.) compared to pup plasma titers (*n* = 14, 19 d.o.). Each point represents an individual dam or pup with each color matched to dam:litter pairs. Dotted horizontal lines represent the limit of detection (1:100) and upper limit of quantification (1:218,700). Samples for which no total anti-RVFV IgG was detected (<1:100) are plotted below the limit of detection. Kruskal-Wallis with Dunn’s multiple comparisons test was used to compare dam plasma and milk total anti-RVFV IgG titers to pup plasma titers (*** indicates *p* = 0.0002, insignificant results not shown). Schematic (**a**) created with BioRender.

We vaccinated pregnant rats at mid-gestation (embryonic day 14, E14) with 1 ×10^5^ PFU RVFV-delNSs/NSm. We chose this gestational age for consistency based on our prior studies in pregnant rats^27^. Dams did not display clinical signs of disease after vaccination and delivered litters between E21-E23, corresponding to 7–9 days post maternal vaccination (d.p.m.v.) (Fig. 1a).

To determine if anti-RVFV humoral immunity was detectable in milk and transferred to neonates, we first measured the durability and transfer kinetics of “total” anti-RVFV IgG in the milk of nursing dams. “Total” refers to an indirect ELISA assay using lysates from RVFV-delNSs/NSm-infected cells and is representative of the IgG response to all potential viral antigens present in the vaccine. Due to the limited volume of milk produced by the dams during the first several days following parturition, our earliest possible milk sampling time corresponded to 12 d.p.m.v., which is neonatal age 3 days old (d.o.) (Fig. 1a). Each dam had detectable total anti-RVFV IgG titers in milk at this earliest timepoint, and titers remained stable until 28 d.p.m.v., right before the pups were weaned (Fig. 1b).

For the neonates, a subset of the pups from each litter were euthanized at the indicated timepoints to measure maternal anti-RVFV IgG in their plasma (Fig. 1a). Roughly 50% of the offspring sampled immediately after delivery (0 d.o., 9 d.p.m.v.; *n* = 13) had low plasma anti-RVFV IgG titers (Fig. 1c). Antibody titers at delivery were not litter-dependent, and it is unclear whether the source of the maternal antibody at this earliest timepoint is from transplacental transfer *in utero* or early colostrum. As pups nursed, total anti-RVFV IgG titers increased and peaked at 19 d.o. (28 d.p.m.v.) (Fig. 1c).

At weaning (22 d.o.; 31 d.p.m.v.), the pups were large enough to permit serial bleeds, so we tested the durability of maternally-acquired total anti-RVFV IgG in longitudinal samples from the remaining offspring (*n* = 5) after weaning. We observed a steady decrease in the total anti-RVFV IgG plasma titers over time after weaning. However, all 5 remaining pups from two different litters had detectable anti-RVFV IgG antibodies in their plasma for at least 21 days post weaning (Fig. 1c). At their peak (19 d.o., 28 d.p.m.v.), the anti-RVFV plasma IgG titers of the pups significantly exceeded the milk titers of the dams (***p = 0.0002) indicating that transfer of anti-RVFV antibodies from dam to pup is highly efficient (Fig. 1d). We conclude that maternal anti-RVFV IgG is efficiently transferred from dam to offspring after RVFV-delNSs/NSm vaccination and persists in the offspring’s circulation for at least 21 days post weaning.

### Passively acquired maternal anti-RVFV immunity protects offspring from lethal RVF

**Fig. 2:**
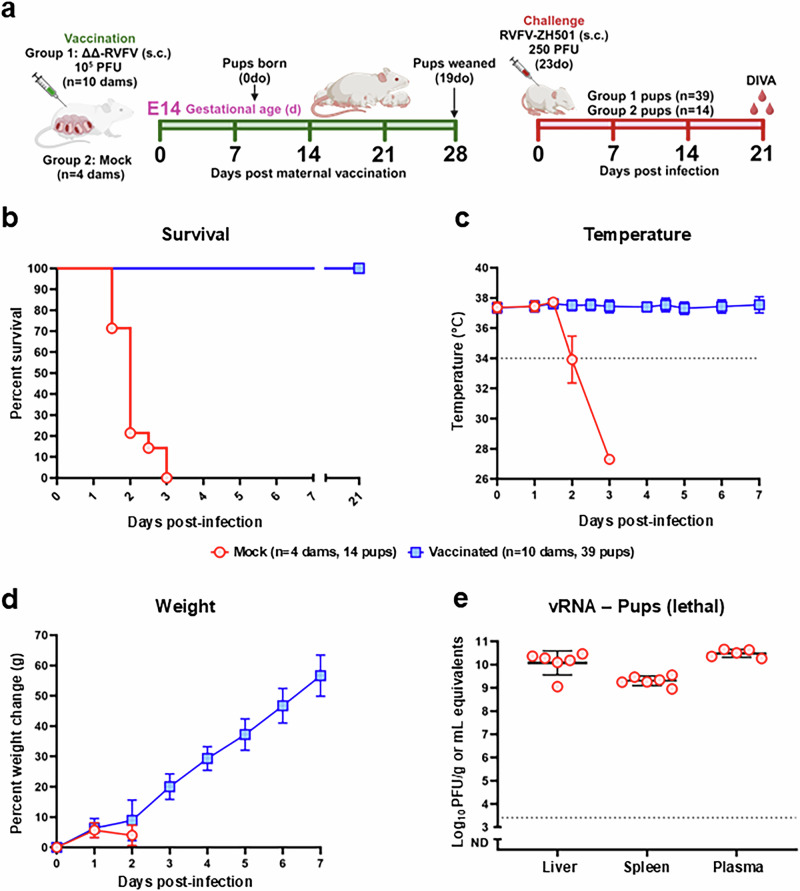
Maternal immunity protects offspring from lethal RVF. **a** Pregnant (E14) dams were vaccinated s.c. with either 1 × 10^5^ PFU RVFV-delNSs/NSm (Group 1, *n* = 10) or media control (Group 2, *n* = 4). Dams delivered and nursed their pups until they reached 19 d.o. (28 d.p.m.v.). At 23 d.o., pups from each group (*n* = 53 total) were inoculated with 250 PFU WT RVFV (ZH501) and monitored daily for clinical signs of disease. Surviving pups were euthanized at 21 dpi to collect blood. **b-d** Survival (**b**), mean body temperature (**c**), and mean percent weight change (**d**) of pups following WT RVFV infection. Red lines and symbols represent pups from mock vaccinated dams (Group 2: *n* = 4 dams, 14 pups) and blue lines and symbols represent pups from RVFV-delNSs/NSm vaccinated dams (Group 1: *n* = 10 dams, 39 pups). Dotted line (**c**) indicates the body temperature threshold for immediate euthanasia criteria. Error bars represent the standard deviation. **e** vRNA in tissue samples collected from a subset (*n* = 6) of lethally infected pups. At the time of necropsy, *n* = 6 pups were selected at random for analysis by qRT-PCR. Tissues from the remaining pups were not tested. One plasma sample from a pup that succumbed to infection could not be collected at the time of necropsy. Schematic (**a**) created with BioRender.

Neonatal animals are exceptionally vulnerable during RVFV outbreaks. We hypothesized that maternal anti-RVFV antibodies acquired by milk could protect offspring from wild-type RVFV challenge during early life. To test this, we vaccinated pregnant dams at E14 with 1 × 10^5^ PFU of RVFV-delNSs/NSm (Group 1) alongside mock vaccinated controls (Group 2). Dams showed no clinical signs of disease after vaccination, delivered their fetuses, and nursed the pups until 19 d.o. (28 d.p.m.v.). At 23 d.o. (32 d.p.m.v.), a total of 39 pups from RVFV-delNSs/NSm vaccinated dams (Group 1 pups) and 14 pups from mock vaccinated dams (Group 2 pups) were challenged subcutaneously with 250 PFU of WT RVFV (ZH501) (Fig. 2a). All pups from mock vaccinated dams succumbed to disease by 3 days post-infection (d.p.i.). In contrast, all pups from RVFV-delNSs/NSm vaccinated dams survived WT RVFV challenge until the study endpoint (21 d.p.i.) (Fig. 2b). Pups born from RVFV-delNSs/NSm vaccinated dams showed no clinical signs of disease and maintained stable body temperature whereas pups from mock vaccinated dams rapidly became hypothermic after challenge (Fig. 2c). All pups from RVFV-delNSs/NSm vaccinated dams gained weight following WT RVFV infection (Fig. 2d). Finally, tissues from a subset of pups (*n* = 6) from unvaccinated dams that succumbed to infection had exceptionally high viral RNA levels within their tissues (Fig. 2e). Together, this demonstrates that the acquisition of maternal anti-RVFV immunity, primarily via milk, is sufficient to completely protect offspring against WT RVFV challenge during early life.

### Maternally acquired anti-RVFV immunity is sufficient to prevent seroconversion against WT RVFV in most pups

Given pups from vaccinated dams were completely protected from lethal disease with a stringent challenge dose of WT RVFV, we used the DIVA (differentiating vaccinated from infected animals) principle to determine if the maternally acquired anti-RVFV immunity prevented seroconversion against WT RVFV. Since RVFV-delNSs/NSm lacks expression of the RVFV-NSs protein, the absence of anti-RVFV-NSs antibodies in surviving pups would indicate a failure to seroconvert against WT RVFV and thus suggests that maternally acquired immunity is potentially sterilizing. Conversely, the presence of anti-RVFV-NSs antibodies would indicate the pups mounted a response to the challenge virus.

**Fig. 3:**
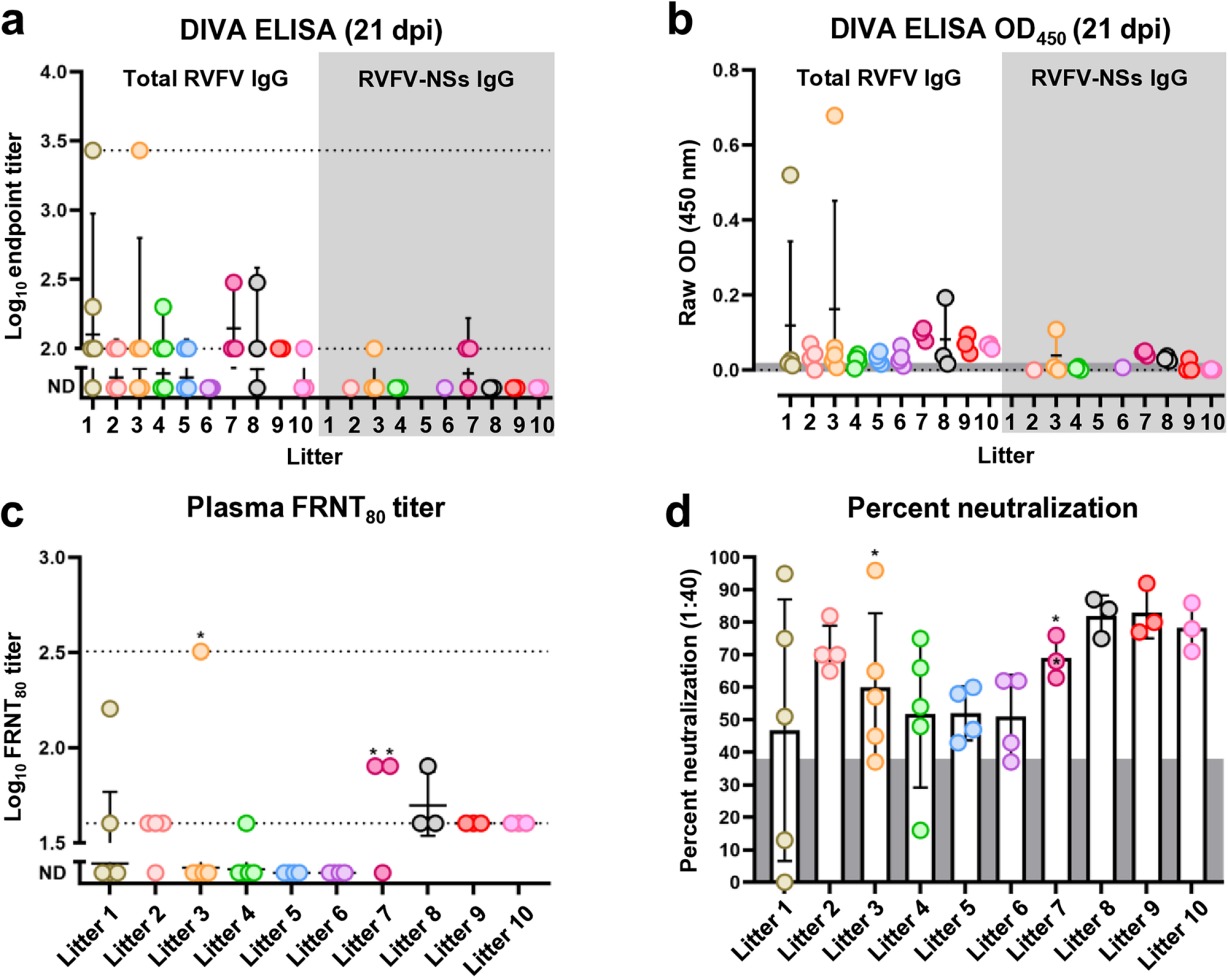
Maternal immune transfer prevents seroconversion to WT RVFV following challenge. **a,** **b** DIVA ELISA performed on plasma collected from surviving pups at 21 dpi (53 d.p.m.v., 44 d.o.). Total anti-RVFV (*n* = 39) or RVFV-NSs IgG (*n* = 20) titers were measured by ELISA and represented as the log_10_ endpoint titer (**a**) or optical density value of the 1:100 dilution read at 450 nm (**b**). Error bars indicate the geometric mean ± the standard deviation (SD) (**a**) or the mean ± SD (**b**). Neutralizing antibody titers (**c**) and percent neutralization (**d**) measured from plasma samples collected from surviving pups at 21 dpi. Asterisks indicate pups that seroconverted to WT RVFV. Lines and bars represent the geometric mean (**c**) or mean (**d**) ±SD. The dotted lines are the limit of detection (1:100 for ELISA, 1:40 for FRNT_80_) and upper limit of quantification (1:2700 for ELISA, 1:320 for FRNT_80_). Samples below the limit of detection are shown as not detected (ND). The shaded line in (**b**) indicates the mean OD_450_ value of all negative control wells. The shaded area in (**d**) represents the average background neutralizing activity of *n* = 10 negative control plasma samples from pups born to mock vaccinated dams. Each point represents an individual pup with each color corresponding to pups within the same litter.

Using plasma collected from the surviving pups at 21 d.p.i. (44 d.o., 53 d.p.m.v.), we performed ELISAs measuring both the total anti-RVFV IgG response against all viral antigens and the anti-NSs IgG response. Of the 39 pups that survived wild-type challenge, 23/39 (59%) had detectable total anti-RVFV IgG at 21 d.p.i. (44 d.o., 53 d.p.m.v.), and most of these were low titers (Fig. 3a, b). For the RVFV-NSs ELISA, we tested plasma from 20 surviving pups across 8 different litters and found that only 3/20 pups had low levels of detectable anti-RVFV-NSs antibodies. Two of the three pups that seroconverted to WT RVFV were from the same litter. However, the entire litter had similar OD_450_ values suggesting the third pup may have seroconverted despite having an endpoint titer below the limit of detection (Fig. 3a, b, litter 7). While the presence of maternal anti-RVFV immunity protected all pups from lethal disease, the fact that some pups (albeit a small number) seroconverted to WT RVFV suggests a threshold antibody titer must be met to prevent seroconversion to WT RVFV and potentially sterilizing immunity.

Results from Fig. 3a,b indicate that maternally acquired immunity is short-lived. While 59% (23/39) of pups still had detectable anti-RVFV IgG by 44 d.o., the titers were low indicating that maternal immunity had largely waned by this time. Despite this, examination of neutralizing capacity at this timepoint revealed that 18 of 39 pups (46%) had FRNT_80_ titers ≥1:40 (Fig. 3c). Given that negative control plasma exhibits low levels of non-specific neutralizing activity, we performed FRNTs on plasma samples from *n* = 10 pups born to mock vaccinated dams and calculated the average, non-specific percent neutralization to be 38% at a dilution of 1:40. Nearly all (34/39) pups born to vaccinated dams had neutralizing activity above background at a plasma dilution of 1:40 (Fig. 3d). Interestingly, compared to RVFV NSs seronegative pups, the 3 pups that seroconverted to RVFV NSs had elevated neutralizing antibody titers (indicated by asterisks), with one neutralizing 96% of virus at 1:40 (Fig. 3c, d).

## Discussion

RVFV poses a significant regional public health threat and continues to cause devastating human and animal disease in Africa. Several vaccines have been developed for veterinary use, and a few have reached human clinical trials. However, none are currently licensed for human use. Live-attenuated RVF vaccines are advantageous as they are generally protective after a single immunization when compared to inactivated vaccines^28^, which is an important consideration in resource-limited regions. A major limitation of historical RVFV LAVs (Smithburn, Clone-13, and MP-12) is they maintain partial virulence in pregnant and/or newborn animals^17–19^. As these animal populations carry the highest disease burden, they likely contribute to increased spillover into humans. Thus, live-attenuated vaccines that are safe for pregnant animals, and enable maternal immune transfer to their offspring, would be highly desirable. Another consideration is that traditional LAVs also lack markers for differentiating infected from vaccinated animals (DIVA). Outbreaks of RVF in livestock often result in the enforcement of trade bans on live animals and animal products from affected regions, resulting in severe socioeconomic damage^29^. Therefore, rationally designed vaccines with serological markers to distinguish natural infection from vaccination is an important consideration to protect livestock owners.

Pre-clinical next-generation RVFV LAVs, such as arMP-12ΔNSm21/384 and RVax-1 have been developed. The arMP-12ΔNSm21/384 vaccine is immunogenic and protective in multiple animal models^30–32^ although poor safety in ewes during early gestation has been reported^31,33^. RVax-1 protects mice from lethal RVF disease^34^; however, no studies have been published to date describing its use in pregnant livestock. Further evaluation regarding the safety and effectiveness of these LAVs during pregnancy would support their use as veterinary RVFV vaccines. Other important RVFV vaccine candidates have recently reached human clinical trials including the four-segmented RVFV LAV, hRVFV-4s, and the chimpanzee adenovirus vectored RVF vaccine, ChAdOx1 RVF^35,36^. Both vaccines have demonstrated a high safety profile in pregnant animal models and protected against RVF^37–39^. RVFV-del-NSs/NSm used in this study is highly immunogenic, DIVA compatible, and a single dose completely protects against RVF in several animal species^20–22^. Our findings indicate that RVFV-delNSs/NSm is an attractive candidate for a maternal vaccination strategy to protect both pregnant animals and their neonatal offspring from RVF.

Maternal vaccination and transfer of immunity has recently gained significant attention with the FDA approval of a maternal RSV vaccine^40^. Vertebrate animals are generally immunologically immature at birth and depend on the transfer of pre-existing maternal immunity for protection during early life^41^. There are two main mechanisms by which maternal antibodies are acquired by offspring, which vary by species. Human infants at term often have higher circulating IgG titers than their mothers, as IgG is efficiently transferred across the hemochorial placenta between 29 and 41 weeks of gestation^42^. In contrast, the synepitheliochorial placenta of ruminants (sheep, cows, and goats) precludes active transport of IgG *in utero*^26^. As a result, newborn ruminants rely on colostrum for maternal immunity during the early post-natal period (24–48 h after birth). In rats, the vast majority of IgG is acquired in the post-natal period through lactation. However, low levels of transplacental maternal antibody transfer still occurs^25,43^. Vaccination at mid-gestation (E14) in our study meant that dams had minimal time (9 days) to mount an immune response prior to delivery. Accordingly, we only detected low maternal anti-RVFV IgG titers in roughly 50% of 0 d.o. (9 d.p.m.v.) pups from vaccinated dams, suggesting milk as the primary source of maternal immunity in this experimental design. Still, maternal immune transfer via milk was rapid and robust as circulating anti-RVFV IgG titers in the pups exceeded maternal titers prior to weaning. The magnitude of maternal antibody transfer may vary depending on timing of vaccination, maternal titers, and age of weaning. As such, these species-specific differences should be considered when designing effective maternal vaccination strategies against RVFV as they may influence both maternal and neonatal vaccine schedules.

In our challenge experiments, maternally acquired immunity provided complete protection against lethal RVF. The majority (85%) of surviving pups assessed by DIVA ELISA had failed to seroconvert against RVFV NSs. Therefore, it is possible that the maternal immunity acquired by these pups was sterilizing. This is an important consideration as surviving pups without sterilizing immunity may be protected from a subsequent infection through a de novo immune response, while those with sterilizing immunity may be susceptible once maternal antibodies wane. A limitation of this analysis is that it is unclear how reliable RVFV NSs is as a marker for DIVA as one study demonstrated inconsistent antibody responses to NSs in RVFV positive livestock^44^. While it is possible that some pups were unable to mount a detectable anti-NSs antibody response, the pups that were seropositive for RVFV NSs also had elevated total anti-RVFV IgG and neutralizing antibody titers compared to NSs seronegative pups. Finally, despite only 59% of surviving pups having low levels of total anti-RVFV IgG present in their plasma by 21 d.p.i. (corresponding to 44 d.o., 53 d.p.m.v.), our data indicate that some offspring from vaccinated dams may still be protected for a longer period due to low levels of neutralizing activity.

This study addresses an important gap in the RVFV vaccine field by measuring the kinetics of maternal antibody transfer in pregnancy and its efficacy against RVF disease in offspring. Mid-gestation maternal vaccination leads to efficient acquisition of anti-RVFV immunity by offspring during the post-natal period via milk. By sampling anti-RVFV antibodies longitudinally in dam milk and pup plasma, we shed light on maternal immune transfer kinetics that could be used to inform optimal vaccine schedules in pregnant livestock. This study is limited by use of a single vaccine dose at one point of gestation. Since ruminants have significantly longer gestational periods than rats, our model may not fully recapitulate the kinetics of maternal antibody transfer in other species. As timing of vaccination is a critical factor governing the effectiveness of maternal immunity, evaluating maternal immune transfer kinetics with lower vaccine doses at different points of gestation or prior to pregnancy is warranted and will be the focus of future studies.

Given RVFV disproportionately affects pregnant and young animals^45^, our finding that maternal immunity fully protects offspring against a stringent challenge dose highlights maternal vaccination as an important strategy to mitigate spread of RVFV. While we hypothesize that anti-RVFV antibodies in milk were the primary source of maternal immunity in our model, it is possible that antibodies acquired *in utero* and/or immune cells present in milk offered some protection to the pups. Therefore, additional studies to identify the mechanistic correlates of protection in our model are necessary.

Finally, while total anti-RVFV IgG titers waned in most pups by the end of our study, roughly half still had minimal detectable neutralizing activity indicating that the protection conferred by maternal immunity may last longer than expected. The durability of anti-RVFV maternal immunity in the weanlings raises two important questions: 1) How long after weaning are pups still protected against RVFV? One study using formalin-inactivated RVFV vaccine in ewes found that low levels of maternally derived neutralizing antibodies (PRNT_50_ 1:10-1:80) persisted in some lambs for 3 months and was partially protective against WT RVFV challenge. However, lambs that were seronegative by 3 months succumbed to infection^46^. Further, passive antibody transfer studies show that neutralizing antibody titers ranging from 1:5-1:20 protect mice from RVF^47^. Therefore, low levels of maternally derived neutralizing antibodies that persist long after weaning may offer some protection against RVFV. 2) Does long-lasting maternal anti-RVFV immunity impair primary vaccination of the neonate? Maternal antibody interference is a phenomenon in which pre-existing maternal immunity blunts both specific and heterologous vaccine-mediated responses. While the mechanism is largely unknown, maternal antibody interference has been described for human and veterinary vaccines^48,49^. As other vaccines have demonstrated the ability to overcome, at least partially, maternal antibody interference^50^, future studies describing the extent of which this occurs for RVFV vaccines are needed.

This study provides insights into the kinetics and protective efficacy of maternal antibodies following vaccination with RVFV-delNSs/NSm. RVFV exemplifies the One Health model as animal, human, and environmental health are inextricably connected. As such, we argue that maternal vaccination of livestock in endemic regions has the potential to reduce livestock mortality, improve agricultural stability, and prevent spillover of RVFV into human populations. Future work aimed toward evaluating anti-RVFV immune transfer after maternal vaccination in relevant ruminant species such as sheep, goats, and cattle would constitute a considerable advancement in the RVFV vaccine field.

## Methods

### Ethics and biosafety

This work complies with all relevant ethical regulations. The animal experiments in this study strictly follow the Guide for the Care and Use of Laboratory Animals of the NIH and Animal Welfare Act. All procedures were approved by the University of Pittsburgh Institutional Animal Care and Use Committee (Protocol #24024312). All work with wild-type RVFV (ZH501) was performed at biosafety level 3 (BSL-3) in the University of Pittsburgh Regional Biocontainment Laboratory (RBL) and followed all safety precautions. The University of Pittsburgh RBL is registered with the Federal Select Agent Program (FSAP) within the Centers for Disease Control and Prevention and the U.S. Department of Agriculture for work with RVFV (ZH501). RVFV-delNSs/NSm is excluded from the Human Health Services and U.S. Department of Agriculture select agents list and all work was performed under BSL-2 conditions per local Institutional Biosafety Committee guidelines.

### Viruses

RVFV (ZH501) used in this study was obtained from Barry Miller (CDC, Ft. Collins, CO) and Stuart Nichol (CDC, Atlanta, GA). RVFV-delNSs/NSm was generated by reverse genetics as previously described^20^. The system was kindly provided by Cesar Albarino, CDC, Atlanta, GA. Virus was propagated using Vero E6 cells (ATCC, CRL-1586) cultured in Dulbecco’s modified Eagle’s medium (DMEM) containing either 2% or 10% FBS, 1% penicillin-streptomycin, and 1% L-glutamine, following standard cell culture procedures. The viral stock titer was determined by viral plaque assay (VPA) as previously described^27^.

### Animals

Time-mated Sprague-Dawley rats (6-10 weeks old) were obtained from Envigo Laboratories (Inotiv). Pregnant rats were delivered to the University of Pittsburgh single-housed in temperature-controlled rooms with 12 h day/12 h night light schedule. Food and water were provided as needed. For all procedures, all rats were anesthetized with vaporized isoflurane (IsoThesia, Henry Schein) and placed on a heating pad (E-Z Systems, HB-163XL) at 37 °C. The data presented in this manuscript are representative of several independent experiments. Sample sizes for all animal experiments were chosen based on our previously published work^27,51,52^.

For maternal immune transfer studies, dams (*n* = 6, E14) were vaccinated subcutaneously (s.c.) in the hind flank with 200 µL of RVFV-delNSs/NSm (1 × 10^5^ PFU) diluted in D2 media (DMEM, 2% (v/v) FBS, 1% penicillin-streptomycin, and 1% L-glutamine). For maternal blood collections, fur was removed from the hind limb (opposite side of vaccination), followed by puncture of the lateral saphenous vein. For milk collections, fur was removed from the abdomen of dams. Dams were then separated from their litters and 4 IU/kg of oxytocin (Fesenius Kabi USA, 912011) was administered intraperitoneally. Milk was expressed manually by gently palpating the mammary tissue. For suckling pups (<22 d.o.), blood was collected by decapitation. When collecting blood from 0 d.o. pups, every attempt was made to collect blood as close to delivery as possible, before the pups began to suckle. However, in some instances pups had already begun suckling at the time of necropsy. For weaned pups (>22 d.o.), blood was collected by lateral saphenous vein puncture. The experimental endpoint for dams was the day of weaning (31 d.p.m.v.). The experimental endpoint for weanlings corresponds to the last blood collection (43 d.o., 52 d.p.m.v.) shown in Fig. 1a. The litter size for each individual dam and the number of pups used at each blood collection timepoint shown in Fig. 1a. are detailed in Table 1. Some pups were excluded due to limited pup availability or asynchronous timepoint collections. Blood at endpoint was collected by cardiac puncture for both dams and weanlings. All blood was collected in ETDA-treated tubes prior to sample processing and storage. All samples were stored at −80 °C for downstream analyses.

**Table 1:**
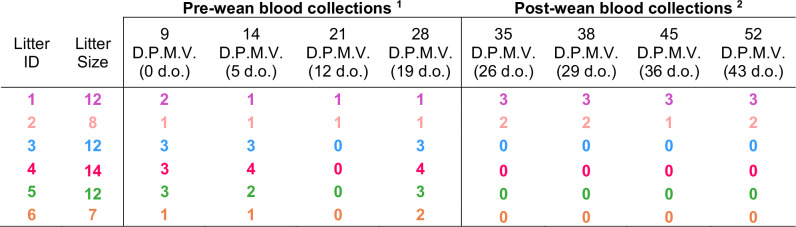
The number of pre- and post-wean pups from RVFV-delNSs/NSm vaccinated dams used for blood samplings at the timepoints shown in Fig. 1c

^1^Pre-wean blood collections were endpoint measurements with different individual pups from each litter being sampled at each timepoint.

^2^Blood collected from pups post-weaning (litter 1, *n* = 3 pups and litter 2, *n* = 2 pups) were serial samplings from the same 5 pups over time to determine the durability of maternal antibodies in the pups after weaning.

Each color corresponds to the dam:litter pairs shown used in Fig. 1.

For WT RVFV (ZH501) challenge studies, dams (*n* = 10, E14) were vaccinated (s.c.) in the hind flank with 200 µL of RVFV-delNSs/NSm (1 × 10^5^ PFU) diluted in D2 media. Concurrently, a control group of dams (*n* = 4, E14) were mock vaccinated with media control (D2). Dams delivered and nursed the pups until 19 d.o. (28 d.p.m.v.), after which pups were weaned and transferred into the RBL (BSL-3). Prior to WT RVFV challenge, pups were implanted with programmable temperature transponders (Avidity Science, TP-1000) s.c. between the shoulders. At 23 d.o., pups were inoculated (s.c) in the hind flank with 200 µL WT RVFV (250 PFU) diluted in D2 media. Body temperature and weight were recorded daily, and pups were monitored twice daily for clinical signs of disease. Animals that met euthanasia criteria were humanely euthanized in accordance with IACUC guidelines. Upon necropsy, liver, spleen and blood were collected and immediately stored at −80 °C for downstream analyses. Blood was collected from surviving pups at the experimental endpoint (21 d.p.i.) shown in Fig. 2. Sex differences were not analyzed as part of this study however, data from both male and female pups were included throughout.

### ELISA

Plasma and milk collected in this study were used for an indirect RVFV whole lysate ELISA^53^. For detection of total anti-RVFV IgG, 96-well MaxiSorp plates (ThermoFisher, 442404) were coated overnight at 4 °C with 100 µL per well of either whole cell lysate from RVFV-delNSs/NSm infected Vero E6 cells or uninfected Vero E6 cells as a negative control. Cell lysates were used at 1:1000 diluted in PBS. For detection of anti-RVFV-NSs IgG, plates were coated overnight at 4 °C with 100 µL per well of either recombinant RVFV-NSs (Custom, bacterially produced, Genscript) or SARS-CoV-2-N protein (generated in house as previously described^54^) as a negative control. Recombinant proteins were used at 2 µg/mL diluted in PBS. Plates were blocked with 5% non-fat, dry milk in PBST (PBS, 0.1% Tween-20) for 1 hr. at 37 °C. Plates were incubated at 37 °C for 2 h. with duplicate samples of either rat plasma or neat milk, serially diluted in block. Rat plasma or neat milk from mock vaccinated animals were used as negative controls. Plates were washed 3 times with PBST and incubated at 37 °C for 1 hr with HRP-labeled goat anti-rat IgG (Invitrogen, 31470), at 1:5000 diluted in block. Plates were washed again three times with PBST and incubated for 10 min. at room temperature with TMB substrate (SeraCare, 5120-0047). Reactions were stopped using TMB Stop Solution (SeraCare, 5150-0021) and plates read at 450 nm. Endpoint titers were defined as the highest plasma dilution with an optical density (OD) exceeding the average of the negative control wells plus two standard deviations after background subtraction of the uninfected, Vero E6 or SARS-CoV-2-N coated plates. If all negative control wells resulted in negative values after background subtraction, three times the standard deviation of the negative control wells was used to define the endpoint titer.

### Focus reduction neutralization assay

Plasma collected in this study was used for a focus reduction neutralization assay^55^. Plasma was heat-inactivated at 56 °C for 30 min., serially diluted in duplicate, and incubated at 37 °C for 1 hr. with 100-200 focus forming units of RVFV-delNSs/NSm-ZSG. The plasma dilutions tested ranged from 1:40 – 1:320. After incubation, 100 µL of the plasma/virus mixture was added to 96-well tissue culture plates containing 2 × 10^4^ Vero E6 cells/well (ATCC, CRL-1586) and incubated 37 °C for 1 h. After incubation, the inoculum was removed and 100 µL 3% carboxymethylcellulose (CMC) (Sigma C4888-500G) was added to each well and incubated 37 °C for ~18 h. CMC was then removed, and wells were washed once with PBS. Plates were then fixed with 4% paraformaldehyde and imaged using a Cytation 5 cell imaging multimode reader (Agilent, Bio Tek). Foci were counted using ImageJ software and the plasma dilution at which 80% virus neutralization occurred was reported as the FRNT_80_. Percent neutralization was determined by comparing plasma-containing wells to control wells containing only virus.

### RNA Isolation and qRT-PCR

Tissue samples were placed in D2 media (w/v) and homogenized. Tissue homogenate or plasma was inactivated by mixing 1:10 in TRIzol reagent (Invitrogen) prior to removal from the RBL. RNA isolation and qRT-PCR were performed as previously described^27^. Quantitation of PFU/mL equivalents was performed using a standard curve generated from an RVFV stock of known titer in PFU/mL.

### Statistics

All graphs and statistical analyses presented in this manuscript were performed using GraphPad Prism. The statistical tests applied to each figure is described in the corresponding figure legends.

## Data Availability

This study did not generate any new sequence or structural datasets. All data generated or analyzed that supports the findings of this study are available from the corresponding author upon request. All materials and unique reagents used in this study are available from the corresponding author upon request.

## References

[1] Hartman, A. Rift Valley Fever. *Clin. Lab Med.* **37**, 285–301 (2017). 28457351 10.1016/j.cll.2017.01.004 PMC5458783

[2] Bird, B. H., Ksiazek, T. G., Nichol, S. T. & Maclachlan, N. J. Rift Valley fever virus. *J. Am. Vet. Med. Assoc.* **234**, 883–893 (2009). 19335238 10.2460/javma.234.7.883

[3] Gerken, K. N. et al. Paving the way for human vaccination against Rift Valley fever virus: A systematic literature review of RVFV epidemiology from 1999 to 2021. *PLoS Negl. Trop. Dis.* **16**, e0009852 (2022). 35073355 10.1371/journal.pntd.0009852 PMC8812886

[4] Gerken, K. N. et al. Urban risk factors for human Rift Valley fever virus exposure in Kenya. *PLoS Glob. Public Health* **2**, e0000505 (2022). 36962424 10.1371/journal.pgph.0000505 PMC10021321

[5] de Glanville, W. A. et al. An outbreak of Rift Valley fever among peri-urban dairy cattle in northern Tanzania. *Trans. R. Soc. Trop. Med Hyg.* **116**, 1082–1090 (2022). 36040309 10.1093/trstmh/trac076 PMC9623736

[6] Pédarrieu, A., Piro-Megy, C., Quellec, J. & Cêtre-Sossah, C. Pasteurization temperatures effectively inactivate Rift Valley fever viruses in milk. *J Virol*, e0202624, 10.1128/jvi.02026-24 (2025). 10.1128/jvi.02026-24 PMC11852957 39882868

[7] Laughlin, L. W., Meegan, J. M., Strausbaugh, L. J., Morens, D. M. & Watten, R. H. Epidemic Rift Valley fever in Egypt: observations of the spectrum of human illness. *Trans. R. Soc. Trop. Med Hyg.* **73**, 630–633 (1979). 575446 10.1016/0035-9203(79)90006-3

[8] Coetzer, J. A. The pathology of Rift Valley fever. I. Lesions occurring in natural cases in new-born lambs. *Onderstepoort J. Vet. Res.* **44**, 205–211 (1977). 613292

[9] Coetzer, J. A. The pathology of Rift Valley fever. II. Lesions occurring in field cases in adult cattle, calves and aborted foetuses. *Onderstepoort J. Vet. Res.* **49**, 11–17 (1982). 7122060

[10] McMillen, C. M. & Hartman, A. L. Rift Valley fever in animals and humans: Current perspectives. *Antivir. Res.* **156**, 29–37 (2018). 29857007 10.1016/j.antiviral.2018.05.009 PMC10316118

[11] Chengula, A. A., Mdegela, R. H. & Kasanga, C. J. Socio-economic impact of Rift Valley fever to pastoralists and agro pastoralists in Arusha, Manyara and Morogoro regions in Tanzania. *Springerplus* **2**, 549 (2013). 24255846 10.1186/2193-1801-2-549 PMC3825084

[12] Kim, Y. et al. The role of livestock movements in the spread of Rift Valley fever virus in animals and humans in Mayotte, 2018-19. *PLoS Negl. Trop. Dis.* **15**, e0009202 (2021). 33684126 10.1371/journal.pntd.0009202 PMC7939299

[13] Ahmad, K. More deaths from Rift Valley fever in Saudi Arabia and Yemen. *Lancet* **356**, 1422 (2000). 11052595 10.1016/S0140-6736(05)74068-X

[14] Hassan, O. A., Ahlm, C. & Evander, M. A need for One Health approach - lessons learned from outbreaks of Rift Valley fever in Saudi Arabia and Sudan. *Infect Ecol. Epidemiol.* **4**, 10.3402/iee.v4.20710 (2014). 10.3402/iee.v4.20710 PMC3915885 24505511

[15] Alkan, C., Jurado-Cobena, E. & Ikegami, T. Advancements in Rift Valley fever vaccines: a historical overview and prospects for next generation candidates. *NPJ Vaccines* **8**, 171 (2023). 37925544 10.1038/s41541-023-00769-w PMC10625542

[16] Métras, R. et al. Estimation of Rift Valley fever virus spillover to humans during the Mayotte 2018-2019 epidemic. *Proc. Natl. Acad. Sci. USA* **117**, 24567–24574 (2020). 32929025 10.1073/pnas.2004468117 PMC7533885

[17] Botros, B. et al. Adverse response of non-indigenous cattle of European breeds to live attenuated Smithburn Rift Valley fever vaccine. *J. Med Virol.* **78**, 787–791 (2006). 16628582 10.1002/jmv.20624

[18] Makoschey, B. et al. Rift Valley Fever Vaccine Virus Clone 13 Is Able to Cross the Ovine Placental Barrier Associated with Foetal Infections, Malformations, and Stillbirths. *PLoS Negl. Trop. Dis.* **10**, e0004550 (2016). 27031621 10.1371/journal.pntd.0004550 PMC4816553

[19] Hunter, P., Erasmus, B. J. & Vorster, J. H. Teratogenicity of a mutagenised Rift Valley fever virus (MVP 12) in sheep. *Onderstepoort J. Vet. Res.* **69**, 95–98 (2002). 12092782

[20] Bird, B. H. et al. Rift valley fever virus lacking the NSs and NSm genes is highly attenuated, confers protective immunity from virulent virus challenge, and allows for differential identification of infected and vaccinated animals. *J. Virol.* **82**, 2681–2691 (2008). 18199647 10.1128/JVI.02501-07 PMC2258974

[21] Bird, B. H. et al. Rift Valley fever virus vaccine lacking the NSs and NSm genes is safe, nonteratogenic, and confers protection from viremia, pyrexia, and abortion following challenge in adult and pregnant sheep. *J. Virol.* **85**, 12901–12909 (2011). 21976656 10.1128/JVI.06046-11 PMC3233145

[22] Smith, D. R. et al. Attenuation and efficacy of live-attenuated Rift Valley fever virus vaccine candidates in non-human primates. *PLoS Negl. Trop. Dis.* **12**, e0006474 (2018). 29742102 10.1371/journal.pntd.0006474 PMC5962102

[23] Coalition for Epidemic Preparedness Innovations. *CEPI partners with University of California, Davis to advance a vaccine against potentially deadly Rift Valley fever virus into clinical trials*, https://cepi.net/cepi-partners-university-california-davis-advance-vaccine-against-potentially-deadly-rift-valley (2023).

[24] Pravieux, J. J., Poulet, H., Charreyre, C. & Juillard, V. Protection of newborn animals through maternal immunization. *J. Comp. Pathol.* **137**(Suppl 1), S32–34 (2007). 17559866 10.1016/j.jcpa.2007.04.009 PMC7094439

[25] Pentsuk, N. & van der Laan, J. W. An interspecies comparison of placental antibody transfer: new insights into developmental toxicity testing of monoclonal antibodies. *Birth Defects Res. B Dev. Reprod. Toxicol.* **86**, 328–344 (2009). 19626656 10.1002/bdrb.20201

[26] Barrington, G. M. & Parish, S. M. Bovine Neonatal Immunology. *Vet. Clin. North Am.: Food Anim. Pract.* **17**, 463–476 (2001). 11692503 10.1016/S0749-0720(15)30001-3 PMC7135619

[27] McMillen, C. M. et al. Rift Valley fever virus induces fetal demise in Sprague-Dawley rats through direct placental infection. *Sci. Adv.* **4**, eaau9812 (2018). 30525107 10.1126/sciadv.aau9812 PMC6281433

[28] Rusnak, J. M., Gibbs, P., Boudreau, E., Clizbe, D. P. & Pittman, P. Immunogenicity and safety of an inactivated Rift Valley fever vaccine in a 19-year study. *Vaccine* **29**, 3222–3229 (2011). 21354483 10.1016/j.vaccine.2011.02.037

[29] Peyre, M. et al. A Systematic Scoping Study of the Socio-Economic Impact of Rift Valley Fever: Research Gaps and Needs. *Zoonoses Public Health* **62**, 309–325 (2015). 25256804 10.1111/zph.12153

[30] Morrill, J. C. et al. Immunogenicity of a recombinant Rift Valley fever MP-12-NSm deletion vaccine candidate in calves. *Vaccine* **31**, 4988–4994 (2013). 23994375 10.1016/j.vaccine.2013.08.003 PMC3808170

[31] Morrill, J. C. et al. Safety and immunogenicity of recombinant Rift Valley fever MP-12 vaccine candidates in sheep. *Vaccine* **31**, 559–565 (2013). 23153443 10.1016/j.vaccine.2012.10.118 PMC3534907

[32] Boumart, Z. et al. Safety and immunogenicity of a live attenuated Rift Valley Fever recombinant arMP-12ΔNSm21/384 vaccine candidate for sheep, goats and calves. *Vaccine* **37**, 1642–1650 (2019). 30773401 10.1016/j.vaccine.2019.01.067

[33] Boumart, Z. et al. Safety and immunogenicity of the Rift Valley fever arMP-12 ΔNSm21/384 candidate vaccine in pregnant ewes. *Vaccine: X* **6**, 100070 (2020). 32793877 10.1016/j.jvacx.2020.100070 PMC7415414

[34] Ikegami, T. et al. Evaluations of rationally designed rift valley fever vaccine candidate RVax-1 in mosquito and rodent models. *NPJ Vaccines* **7**, 109 (2022). 36131104 10.1038/s41541-022-00536-3 PMC9492667

[35] Leroux-Roels, I. et al. Safety and immunogenicity of the live-attenuated hRVFV-4s vaccine against Rift Valley fever in healthy adults: a dose-escalation, placebo-controlled, first-in-human, phase 1 randomised clinical trial. *Lancet Infect. Dis.* **24**, 1245–1253 (2024). 39068957 10.1016/S1473-3099(24)00375-X

[36] Jenkin, D. et al. Safety and immunogenicity of a ChAdOx1 vaccine against Rift Valley fever in UK adults: an open-label, non-randomised, first-in-human phase 1 clinical trial. *Lancet Infect. Dis.* **23**, 956–964 (2023). 37060917 10.1016/S1473-3099(23)00068-3 PMC7614834

[37] Wichgers Schreur, P. J., van Keulen, L., Kant, J. & Kortekaas, J. Four-segmented Rift Valley fever virus-based vaccines can be applied safely in ewes during pregnancy. *Vaccine* **35**, 3123–3128 (2017). 28457675 10.1016/j.vaccine.2017.04.024

[38] Wichgers Schreur, P. J. et al. A single vaccination with four-segmented Rift Valley fever virus prevents vertical transmission of the wild-type virus in pregnant ewes. *npj Vaccines* **6**, 8 (2021). 33420095 10.1038/s41541-020-00271-7 PMC7794363

[39] Stedman, A. et al. Safety and efficacy of ChAdOx1 RVF vaccine against Rift Valley fever in pregnant sheep and goats. *NPJ Vaccines* **4**, 44 (2019). 31646004 10.1038/s41541-019-0138-0 PMC6802222

[40] U.S. Food & Drug Administration. *FDA Approves First Vaccine for Pregnant Individuals to Prevent RSV in Infants*, https://www.fda.gov/news-events/press-announcements/fda-approves-first-vaccine-pregnant-individuals-prevent-rsv-infants (2023).

[41] Hasselquist, D. & Nilsson, J. A. Maternal transfer of antibodies in vertebrates: trans-generational effects on offspring immunity. *Philos. Trans. R. Soc. Lond. B Biol. Sci.* **364**, 51–60 (2009). 18926976 10.1098/rstb.2008.0137 PMC2666691

[42] Palmeira, P., Quinello, C., Silveira-Lessa, A. L., Zago, C. A. & Carneiro-Sampaio, M. IgG placental transfer in healthy and pathological pregnancies. *Clin. Dev. Immunol.* **2012**, 985646 (2012). 22235228 10.1155/2012/985646 PMC3251916

[43] Appleby, P. & Catty, D. Transmission of immunoglobulin to foetal and neonatal mice. *J. Reprod. Immunol.* **5**, 203–213 (1983). 6620251 10.1016/0165-0378(83)90236-x

[44] Fernandez, J.-C. et al. The Nonstructural Protein NSs Induces a Variable Antibody Response in Domestic Ruminants Naturally Infected with Rift Valley Fever Virus. *Clin. Vaccin. Immunol.* **19**, 5–10 (2012). 10.1128/CVI.05420-11 PMC3255954 22072723

[45] Ikegami, T. & Makino, S. The pathogenesis of Rift Valley fever. *Viruses* **3**, 493–519 (2011). 21666766 10.3390/v3050493 PMC3111045

[46] Harrington, D. G. et al. Evaluation of a formalin-inactivated Rift Valley fever vaccine in sheep. *Am. J. Vet. Res* **41**, 1559–1564 (1980). 7224281

[47] Watts, D. M. et al. Estimation of the Minimal Rift Valley Fever Virus Protective Neutralizing Antibody Titer in Human Volunteers Immunized with MP-12 Vaccine Based on Protection in a Mouse Model of Disease. *Am. J. Trop. Med Hyg.* **107**, 1091–1098 (2022). 36122681 10.4269/ajtmh.22-0356 PMC9709016

[48] Englund, J. A. et al. The effect of maternal antibody on the serologic response and the incidence of adverse reactions after primary immunization with acellular and whole-cell pertussis vaccines combined with diphtheria and tetanus toxoids. *Pediatrics* **96**, 580–584 (1995). 7659480

[49] Knuutila, A. et al. Effect of immunization during pregnancy and pre-existing immunity on diphtheria-tetanus-acellular pertussis vaccine responses in infants. *Emerg. Microbes Infect.* **12**, 2204146 (2023). 37060181 10.1080/22221751.2023.2204146 PMC10161941

[50] England, R. N. et al. Evaluation of mRNA-LNP and adjuvanted protein SARS-CoV-2 vaccines in a maternal antibody mouse model. *npj Vaccines* **9**, 110 (2024). 38890316 10.1038/s41541-024-00901-4 PMC11189435

[51] McMillen, C. M. et al. Congenital Rift Valley fever in Sprague Dawley rats is associated with diffuse infection and pathology of the placenta. *PLoS Negl. Trop. Dis.* **16**, e0010898 (2022). 36315601 10.1371/journal.pntd.0010898 PMC9648853

[52] McMillen, C. M. et al. A highly potent human neutralizing antibody prevents vertical transmission of Rift Valley fever virus in a rat model. *Nat. Commun.* **14**, 4507 (2023). 37495594 10.1038/s41467-023-40187-z PMC10372071

[53] McElroy, A. K., Albariño, C. G. & Nichol, S. T. Development of a RVFV ELISA that can distinguish infected from vaccinated animals. *Virol. J.* **6**, 125 (2009). 19678951 10.1186/1743-422X-6-125 PMC2733132

[54] Xu, L. et al. A Cross-Sectional Study of SARS-CoV-2 Seroprevalence between Fall 2020 and February 2021 in Allegheny County, Western Pennsylvania, USA. *Pathogens* **10**, 10.3390/pathogens10060710 (2021). 10.3390/pathogens10060710 PMC8226606 34204122

[55] Harmon, J. R., Barbeau, D. J., Nichol, S. T., Spiropoulou, C. F. & McElroy, A. K. Rift Valley fever virus vaccination induces long-lived, antigen-specific human T cell responses. *npj Vaccines* **5**, 17 (2020). 32140261 10.1038/s41541-020-0166-9 PMC7048758

